# Mid-infrared passively switched pulsed dual wavelength Ho^3+^-doped fluoride fiber laser at 3 μm and 2 μm

**DOI:** 10.1038/srep10770

**Published:** 2015-06-04

**Authors:** Jianfeng Li, Hongyu Luo, Lele Wang, Yong Liu, Zhijun Yan, Kaiming Zhou, Lin Zhang, Sergei K. Turistsyn

**Affiliations:** 1 State Key Laboratory of Electronic Thin Films and Integrated Devices, School of Optoelectronic Information, University of Electronic Science and Technology of China (UESTC), Chengdu 610054, China; 2 Institute of Photonic and Technology (AIPT), Aston University, Birmingham, UK; 3 Jiangsu Key Laboratory of Medical Optics, Suzhou Institute of Biomedical Engineering and Technology, Chinese Academy of Sciences, Suzhou 215163, China

## Abstract

Cascade transitions of rare earth ions involved in infrared host fiber provide the potential to generate dual or multiple wavelength lasing at mid-infrared region. In addition, the fast development of saturable absorber (SA) towards the long wavelengths motivates the realization of passively switched mid-infrared pulsed lasers. In this work, by combing the above two techniques, a new phenomenon of passively Q-switched ~3 μm and gain-switched ~2 μm pulses in a shared cavity was demonstrated with a Ho^3+^-doped fluoride fiber and a specifically designed semiconductor saturable absorber (SESAM) as the SA. The repetition rate of ~2 μm pulses can be tuned between half and same as that of ~3 μm pulses by changing the pump power. The proposed method here will add new capabilities and more flexibility for generating mid-infrared multiple wavelength pulses simultaneously that has important potential applications for laser surgery, material processing, laser radar, and free-space communications, and other areas.

Mid-infrared (mid-IR) lasers, generally defined as having an operation wavelength in the spectral region of 2 ~ 20 μm, have attracted tremendous scientific and technological interests owing to their widespread applications^1^. This wavelength range not only contains some strong vibration transitions of many important molecules, thus, exhibiting enormous potential in medical, spectroscopy, chemical and biomolecular sensing, but also covers the atmospheric transmission windows of 3 ~ 5 μm and 8 ~ 13 μm which are important for defense, atmospheric, security and industrial applications^1^. Currently, several diverse approaches including solid-state lasers^2^, Quantum cascade lasers (QCLs)^3^, difference frequency generation (DFG)^4^, sum frequency generation (SFG)^4^ and optical parametric oscillator (OPO)^5^ have been employed to generate the emission at different subranges of the mid-IR wavelength region. With the development of infrared glass fiber, mid-IR fiber lasers are attracting increasing attentions because they possess some inherent merits such as excellent beam quality, good heat dissipation, high efficiency and compact packaging compared to the previously mentioned sources^6^, though certainly, they cannot be regarded as a full replacement for the other lasers and rather provide complimentary capabilities. Recent research on mid-IR fiber lasers has mainly been focused at the short mid-IR regime (2 ~ 4 μm) due to the limitation of current host materials and dopant technology^7^,^8^,^9^,^10^,^11^,^12^,^13^,^14^,^15^,^16^. Tm^3+^, Ho^3+^, and Er^3+^are main dopant trivalent rare earth ions in mid-IR fiber lasers, specifically Tm^3+^produces gain around 2.0 and 2.3 μm^7^,^8^, Ho^3+^produces gain at about 2.1, 2.8, 3.0, 3.2, and 4.0 μm^9^,^10^,^11^,^12^,^13^, and Er^3+^produces gain at ~2.9 and 3.5 μm^14^,^15^,^16^. Therefore, the emphasized ~2 μm and ~3 μm fiber lasers especially operating with μs- or ns-wide pulses display enormous potential in some areas such as plastic and polymer processing, laser scalpels, non-invasive medical diagnosis, infrared countermeasures, and pumping OPOs and others. So far, laser operation in this waveband has been achieved by exploiting one of the following two techniques: the first is Q-switching, in particular, passively Q-switching, that is attractive owing to its simple structure and low loss, in which the cavity loss is modulated using a material saturable absorber (SA)^17^,^18^,^19^,^20^,^21^,^22^,^23^ e.g., single-wall carbon nanotubes (SWCNTs)^17^, graphene^18^,^19^, a transition metal doped crystal (e.g., Fe^2+^:ZnSe, Cr^2+^:ZnSe, etc.)^20^,^21^, or a semiconductor saturable absorber mirror (SESAM)^22^,^23^; the second is gain-switching in which the pulsed pumping was employed to modulate the cavity gain^24^,^25^.

In the past few years, a variety of passively Q-switched and gain-switched pulsed fiber lasers emitting at ~2 μm and ~3 μm have been reported. Since the first demonstration of SWCNTs-based pulsed fiber laser at 1.55 μm in 2003^26^, its controllable absorption band has been successfully exploited to Q-switching of ~1 μm and ~2 μm band lasers^17^,^27^, but has failed at ~3 μm due to the difficulty in achieving bandgap control at the longer wavelengths. Graphene, as a novel fashionable two-dimensional (2D) material, exhibits a prominent broadband saturable absorption property owing to its zero bandgap structure^28^. Graphene has also been exploited in Q-switching ~2 μm and ~3 μm fiber lasers^18^,^19^,^29^,^30^. However, the low ratio of modulation depth to total transmission as well as the intrinsic ultrafast recovery time is more suitable for mode-locking^31^. The Cr^2+^:ZnSe and Fe^2+^:ZnSe crystal as SAs exhibit strong absorption at ~2 μm and ~3 μm, respectively, but they have the disadvantages of being bulk structures requiring complex confocal alignment^20^,^21^,^30^,^32^. SESAM, as a mature SA, is constantly improving through innovations. It has a remarkably excellent performance as well as the ability of customize some of its parameters, i.e., modulation depth, non-saturable loss, recovery time, etc., mainly attributing to well-developed semiconductor technologies such as bandgap and defect engineering and growth. This technology is widely available in the commercial market despite the relatively complex fabrication process which is required. SESAM has been widely applied into ~2 μm Q-switching^22^,^33^,^34^ and recently into ~3 μm Q-switching where a specifically structured SESAM whose InAs absorber layer was sandwiched between an Au-coated mirror and a GaAs wafer^23^. This new structure was designed to reduce the fabrication time and material consumption while improving the damage threshold and feedback band thus providing the feasibility of broadband Q-switching.

As another alternative for pulse generation, gain switching has already exhibited great potential at both ~2 μm and ~3 μm bands^24^,^25^,^35^,^36^. Some recent pivotal demonstrations, i.e. in-band fast gain switching based on short pulses^24^, hybrid-pumping exploiting both continuous wave (CW) and pulse components^35^ and overcoming unstable relaxation oscillation in conventional gain switching have also emphasized the importance of this technique.

Although mid-IR pulsed fiber lasers at ~2 μm and ~3 μm have experienced fast development, most of them only emitted at single wavelength due to excitation of only one transition^17^,^18^,^19^,^20^,^21^,^22^,^23^,^24^,^25^,^27^,^29^,^30^,^32^,^33^,^34^,^35^,^36^. Pulsed lasers capable of simultaneously generating ~2 μm and ~3 μm emissions would undoubtedly exhibit more advantages (e.g., flexibility, convenience, high efficiency, etc.) in some practical applications where the wavelength is alternatively required or both are needed simultaneously. Laser surgery is a typical example as both wavelengths are located at the absorption peaks of water molecules^37^. Specifically, ~2 μm pulses have exhibited excellent performance in spinal surgery (e.g., laser diskectomy, laser foraminoplasty)^38^, ulrolog (e.g., lithotripsy)^39^,^40^, and arthroscopy (e.g., capsular shrinkage, cartilage smoothing, miniscectomy, synovectomy)^38^, while ~3 μm pulses have superiority in cutaneous surgery^41^, bone surgery (e.g., oral surgery, implant dentistry)^42^, brain tissue treatment^43^, and ophthalmology^44^. Therefore, the dual wavelength pulsed components can be alternatively selected according to the practical requirement. Besides, in some surgeries of tissue ablation, the ~2 μm and ~3 μm lasers at either CW^45^ or pulsed^46^,^47^,^48^ state are also simultaneously required owing to the improved ablation efficiency and reduced heat diffusion zone. Moreover, material processing is another area that could benefit from dual wavelength sources selectable. Better performance in the processing of plastics has been demonstrated using a ~2 μm instead of visible/near-infrared or CO_2_ pulsed laser as a result of its moderate absorption by plastics^49^, while ~3 μm pulses are a good candidate for cleaning of painted surfaces due to strong absorption by OH bonds at 2.94 μm^50^. Other potential applications include free-space telecommunications, laser imaging radar and range finding which can all be carried out using either ~2 μm or ~3 μm pulses owing to both wavelength locating within the atmospheric transparency windows of 2.05~2.5 μm and 3~5 μm, respectively. Dual wavelength synchronously operation could facilitate the expansion of information propagation capability by increasing the number of channels and could enhance measurement accuracy as a result of double detections. Therefore, the availability of integrated dual wavelength ~2 μm and ~3 μm pulsed fiber sources can provide greater flexibility, convenience and selectivity for these applications and is also an interesting research topic.

Fortunately, the lower cascade transitions (i.e., ^5^I_7_ → ^5^I_8_ and ^5^I_6_ → ^5^I_7_) of Ho^3+^-doped ZBLAN fiber pumped by ~1150 nm are well-sitting in ~2 μm and ~3 μm emission bands^11^ thus providing the possibility of dual wavelength pulsing. Recently, we have utilized an acousto-optic modulator (AOM) to actively Q-switch cascade transitions and subsequently to Q-switch one transition thus inducing adjacent transition gain switching. Stable dual wavelength pulsing (i.e., ~2 μm and ~3 μm) was obtained in both cases^51^,^52^. However, the employment of external driving AOM undoubtedly removes the intrinsic compactness and simplicity of fiber lasers.

In this paper, using a specifically designed SESAM optimized for ~3 μm, we report the first investigation of a dual wavelength, passively switched, cascade pulsed Ho^3+^-doped fluoride fiber laser. The results have confirmed the feasibility of simultaneously generating ~2 μm and ~3 μm passively switched pulses from a shared oscillator. In addition, the characteristics of the dual wavelength pulses, i.e., repetition rate, pulse width and time delay, have been investigated and analyzed in detail.

## Results

**Figure 1:**
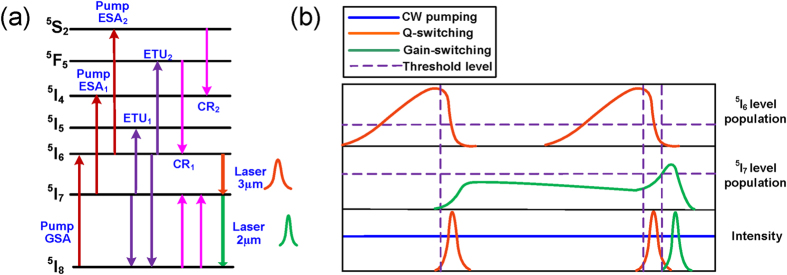
(**a**) Simplified energy level of cascade Ho^3+^-doped fluoride fiber, (**b**) schematic illustration of dual wavelength pulses of laser upper levels populations and temporal domain evolution.

**Figure 2:**
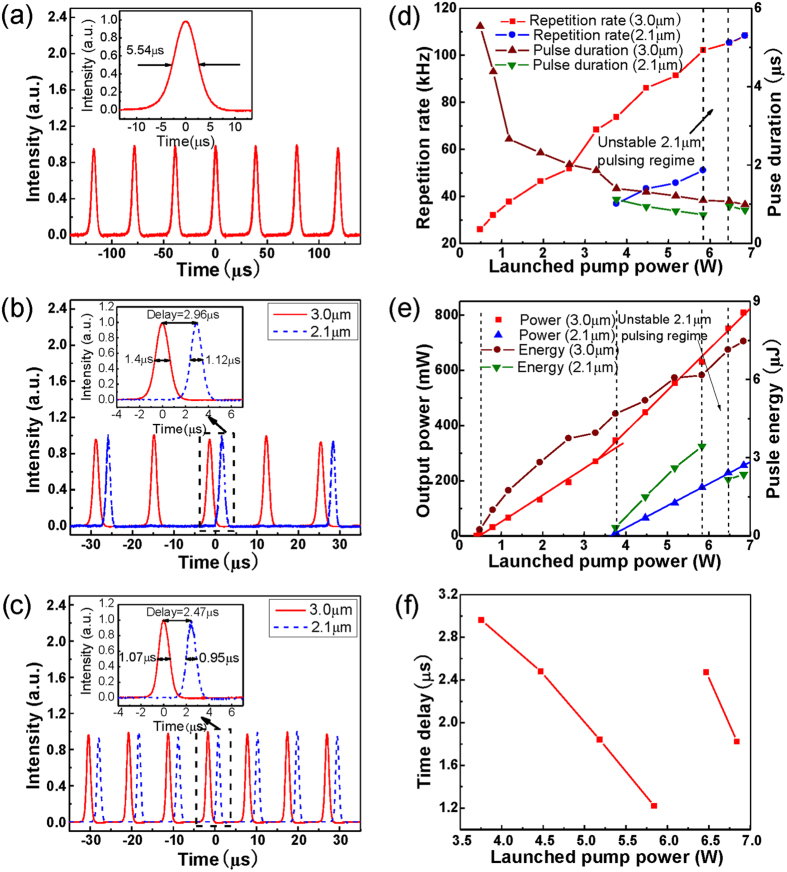
Pulse time domain waveforms recorded by digital oscilloscope at the launched pump power of **(a) 0.48 W**, (b) 3.76 W, (c) 6.47 W; (**d**) Repetition rates and pulses durations (**e**) output powers and pulse energies of both ~3 μm and ~2 μm pulses and their (**f**) time delays as a function of launched pump power.

The passively switched, dual wavelength laser was designed and constructed as described in the Methods part. In this section, the performances of each output band are studied as a function of the pump levels. As the launched pump power was increased to 0.41 W, only CW ~ 3 μm laser initiated from broadband ASE operation was observed as a result of a low laser threshold for ^5^I_6_ → ^5^I_7_ transition, as shown in Fig. 1(a). At this pump level, no sign of pulsing can be observed indicating that the intra-cavity ~3 μm emission intensity was not high enough to activate the nonlinear absorption of the SESAM. Once the pump power was increased to 0.48 W, a stable ~3 μm Q-switched pulse train was observed in temporal domain as depicted in Fig. 2(a), albeit with a low SNR of ~33 dB. The relatively low repetition rate of 26 kHz and large pulse duration of 5.54 μs were mainly due to the low upper level ^5^I_6_ population accumulation rate and the small intra-cavity net gain coefficient at this low pump level. Further increasing the pump level to 3.76 W, stable ~3 μm Q-switched pulses were maintained with a significantly improved SNR of >40 dB. In this pump power range (0.48 W ~ 3.76 W), the repetition rate increased from 26 kHz to 73.7 kHz and the pulse duration decreased from 5.5 μs to 1.4 μs, as shown in Fig. 2(d). This is because of the faster population buildup and higher energy storage on ^5^I_6_ level. As shown in Fig. 2(e), the output power increased near linearly from 6.1 mW to 345.2 mW at a slope efficiency of 9.76% while the pulse energy increased from 0.23 μJ to 4.68 μJ.

**Figure 3:**
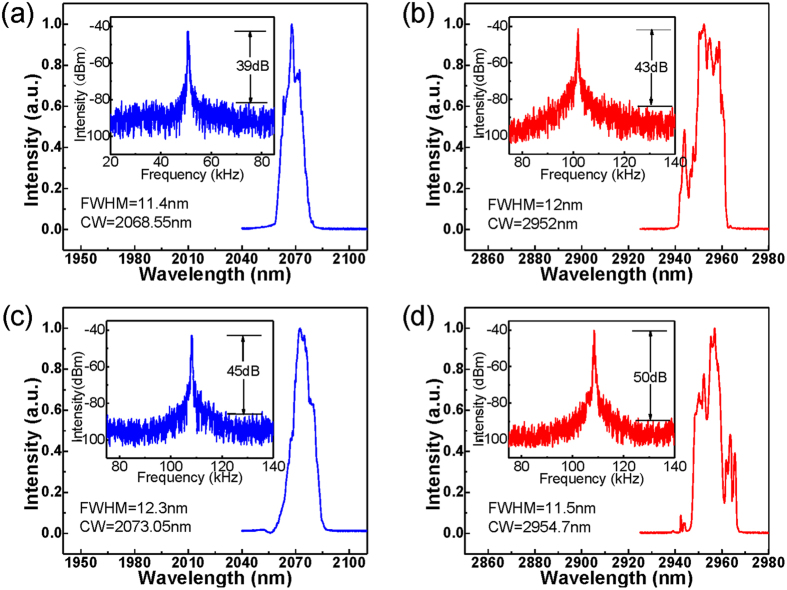
Optical and RF spectra for dual wavelength pulsed components at the launched pump power of (a) (b) 3.76 W and (c) (d) 6.47 W.

Once the pump power was increased above 3.76 W, the passively switched the ~2 μm pulses emitted from the ^5^I_7_ → ^5^I_8_ transition were observed as shown in Fig. 3(b), while stable ~3 μm Q-switched pulses were still maintained. Their optical and RF spectra were measured giving a center wavelength and SNR of 2068.55 nm and ~39 dB for the ~2 μm pulses, and 2952.0 nm and ~43 dB for the ~3 μm pulses, respectively, as shown in Fig. 3(a,b). The ~2 μm pulses were confirmed to be the gain-switched pulses rather than Q-switched pulses as will be discussed below. It is also observed from Fig. 2(b) that the ~2 μm pulse train has halved the repetition rate of the ~3 μm pulse train and displayed a μs level time delay (specifically 2.96 μs at this pump level) relative to the ~3 μm pulse train. The discrepancy in the repetition rate between the ~3 μm and ~2 μm pulses was mainly due to not achieving the sufficient populations for ^5^I_7_ level with only one ~3 μm Q-switched pulse at low pump power levels. Moreover, the time delay between each ~2 μm pulses and its adjacent ~3 μm pulse should be noted, since this was not equal to the typical buildup time of a gain switched pulse. In this case (see Fig. 2(b)), each temporal domain period includes two ~3 μm pulses and one ~2 μm pulse. As illustrated in Fig. 1(b), the first ~3 μm pulse was not strong enough to induce populations on ^5^I_7_ level to arrive at the laser threshold level, however, the population on the ^5^I_7_ level was accumulated continuously. During the time interval between the two ~3 μm pulses, the population on ^5^I_7_ level was partly consumed by some parasitic processes i.e., energy transfer upconversion (ETU_1_) and excited-state absorption (ESA_1_), as shown in Fig. 1(a). Fortunately, the ETU_1_ process has only a small contribution owing to the low transition rates in a low Ho^3+^dopant concentration system^53^. Moreover, the ESA_1_ process will only decrease the population on ^5^I_7_ level slightly as a result of the low pumping rate in this cladding pump system^53^. With the appearance of the second ~3 μm pulse, the population on the ^5^I_7_ level will continue to be increased and consequently exceeded the required level for the ~2 μm laser. As shown in Fig. 2(d), the repetition rate and pulse duration of ~3 μm pulses were increased to 102.1 kHz and reduced to 1.1 μs respectively as the pump power was increased to 5.84 W. This behavior is typical of Q-switched pulses. The corresponding gain-switched ~2 μm component displayed a synchronized evolution with its pump signal. The higher repetition rate and narrower pulse duration of the ~3 μm pulses lead to faster pumping of the ^5^I_7_ level, thus accelerating the formation of ~2 μm pulses. Hence, its repetition rate increased from 36.8 kHz to 51.1 kHz and the pulse duration decreased from 1.12 μs to 0.73 μs as the pump power was increased from 3.76 W to 5.84 W. Furthermore, the pulse duration of the ~2 μm pulses was narrower than their pump signals, i.e., Q-switched ~3 μm pulses, and this is typical of a gain switched feature^24^,^25^. As shown in Fig. 2(e), the output power of the ~3 μm pulses increased to 629.2 mW with an improved slope efficiency of 14.95% when the pump power was increased to 5.84 W while their pulse energy increased to 6.16 μJ. This is because of the cascaded ~2 μm lasing which rapidly depletes the population on the lower laser level of the ^5^I_6_ → ^5^I_7_ transition. Note that the increased energy of ~3 μm pulses also results in faster population accumulation on the ^5^I_7_ level thus narrowing the ~2 μm pulse. In a similar way to ~3 μm pulse, the pulse power for the ~2 μm components was observed to increase from 11.7 mW to 175.5 mW at a slope efficiency of 8.01% and the pulse energy increased from 0.32 μJ to 3.44 μJ. Furthermore, the time delay between the ~2 μm pulse and its adjacent ~3 μm pulse decreased to 1.22 μs when the pump power was increased to 5.84 W, as shown in Fig. 2(f), indicating that the high pumping rate accelerated the formation of ~2 μm pulses.

Once this pump level was exceeded, stable ~3 μm Q-switched pulses but with following unstable ~2 μm pulses were observed. The ~2 μm pulse train exhibited severe timing jitter and its repetition rate jumped between a half and matching that of the ~3 μm pulse train suggesting the ~2 μm pulses operated at a threshold state in which the inversion population for ~2 μm transition was induced by two ~3 μm pulses or one ~3 μm pulse as a result of the increasing ~3 μm pulse energy. Fluctuation of the energy and duration of ~3 μm pulse caused system to switch between these two states, and consequently led to the repetition rate jumping.

When the pump power was increased to 6.47 W, stable ~2 μm pulses were observed again with their repetition rate matched to the ~3 μm pulses, as show in Fig. 2(c). Their optical and RF spectra were measured as shown in Fig. 3(c,d) exhibiting slightly red-shifted center wavelengths of 2073.05 nm and 2954.7 nm and increased SNRs of ~45 dB and ~50 dB, respectively. Compared to the pulses generated at a pump power of 5.84 W, the energy of the ~2 μm pulses fell from 3.44 μJ to 2.17 μJ as a result of its doubled repetition rate. Its duration also jumped from 0.73 μs to 0.95 μs resulting from the decreased accumulated populations on ^5^I_7_ level. The increased time delay from 1.22 μs to 2.47 μs as shown in Fig. 3(f) suggests that a longer build-up time (calculated from its adjacent ~3 μm pulse) of ~2 μm pulse was needed for population at threshold level to be induced by a single ~3 μm pulse instead of two. Stable ~2 μm and ~3 μm pulses were maintained when the pump power was increased to the maximum of 6.84 W. At this point, the maximum output power of 808.9 mW and pulse energy of 7.47 μJ were achieved for the ~3 μm pulses with a repetition rate of 108.34 kHz and a pulse duration of 0.99 μs, while maximum output power of 255.3 mW and pulse energy of 2.17 μJ were obtained for ~2 μm pulses with a repetition rate of 108.29 kHz and a pulse duration of 0.85 μs. Meanwhile, the time delay between adjacent ~2 μm and ~3 μm pulses was further shortened to 1.82 μs.

In the demonstrated dual wavelength laser system, the ~2 μm and ~3 μm pulses shared the same cavity formed by the broadband SESAM and a perpendicularly cleaved fiber end. In order to determine if the cascaded ~2 μm pulses were Q-switching or gain-switching, two extra experiments were subsequently performed. Firstly, we designed an experimental setup for generating Q-switched ~3 μm induced ~2 μm gain switched pulse. A dichroic mirror labeled C with >90% transmission at ~3 μm and ~87% reflection at ~2 μm was placed between the SESAM and the dichroic mirror A. Thus, the laser cavities were partly altered; the ~3 μm laser cavity was still defined by the SESAM and the perpendicularly cleaved fiber end while the dichroic mirror C only acts as a linear loss mechanism; however the ~2 μm laser cavity was terminated by the dichroic mirror C instead of the SESAM. These new composite cavities prevented ~2 μm emission from being modulated by the SESAM thus excluding the possibility of SESAM Q-switched ~2 μm emission. In this case, the altered laser produced similar pulse evolution of ~2 μm pulses with almost identical repetition rate to that displayed in Fig. 3(b,c) though with slightly decreased laser thresholds. Therefore, the previously produced ~2 μm pulses appeared to be generated by gain switching; although there remains the small possibility of SESAM Q-switching. Accordingly, another experiment was performed where the dichroic mirror C was replaced by a dichroic mirror labeled D with ~90% transmission at ~2 μm and >95% reflection at ~3 μm. Thus the cavities of the ~2 μm and ~3 μm emissions were exchanged, i.e., the ~2 μm and ~3 μm emissions were terminated by the SESAM and the dichroic mirror D, respectively. In this case, the SESAM will provide more feedback for the ~2 μm emission compared to the residual reflection from dichroic mirror D and 8^0^ cleaved fiber end. However, over the whole available pump range, the dual wavelength components were essentially operated in a CW state. This was mainly due to the wavelength related saturable influence of the SESAM and the power level of ~2 μm emission. The measured saturable influence of 1771 μJ/cm^−2^ at ~1 μm and 70 μJ/cm^2^ at ~3 μm^23^, indicate that the saturable influence decreases dramatically with increasing wavelength. Thus, the saturable influence at ~2 μm should be much larger than that at ~3 μm. On the other hand, the power level of ~2 μm emission is lower than that of ~3 μm emission at a same pump level resulting from cascading induced higher threshold. Therefore, the saturable fluence of the SESAM at ~2 μm was not reached and consequently the ~2 μm Q-switched pulses were not observed. However, the ~2 μm Q-switching should be also possible if the power of ~2 μm emission can be further improved by increasing the pump power and decreasing the threshold. Therefore this also excludes the possibility of SESAM Q-switched ~2 μm emission in the original shared cavity. According to the above experiments, the original cascade ~2 μm passively switched pulses were verified to be ~2 μm gain switched components which were induced by the ~3 μm passively Q-switched pulses.

## Discussion

In this section, further performance of the demonstrated passively switched cascade dual wavelength pulsed fiber laser in terms of pulse repetition rates, time delay, durations and energies will be discussed.

Firstly, we will discuss the repetition rates of the dual wavelength components. Here, we found that the ~2 μm gain switched pulses operated at either the half or at the same repetition rate as the ~3 μm Q-switched pulses, depending on the pump power level. No ~2 μm pulses at one third or even quarter repetition rate of ~3 μm pulses was observed at lower pump level due to the limitation of ~2 μm laser threshold. Therefore, more temporal operation modes could be expected if the ~2 μm laser threshold was further reduced by for example: lowering the Ho^3+^ ions dopant concentration while maintaining sufficient ~3 μm gain^54^, or increasing the ~2 μm emission feedback. However, the laser will consequently have the same repetition rate in both wavebands when the pump power increase to or above the threshold level. Moreover, it is predicted that the oscillation relaxation or multiple pulsing in each ~2 μm pulse period could occur at very high pump levels where the populations on ^5^I_7_ level provided by one ~3 μm pulse can not be depleted by one ~2 μm pulse.

Secondly, the time delay between ~2 μm and ~3 μm pulses could be a critical parameter concerned in some practical applications and appears strongly related to the population accumulation rate on the ^5^I_7_ level. Accordingly, the transition rates of all processes relating to the ^5^I_7_ level including ^5^I_6_ → ^5^I_7_ transition, ESA_1_, ETU_1_, CR_1_ and CR_2_ will have an impact on the time delay, i.e., more supply and less depletion for this level will result in a shorter time delay. Owing to the comparative low ESA_1_, ETU_1_, CR_1_ and CR_2_ rate in low-concentration cladding pumped cascade system, ^5^I_6_ → ^5^I_7_ transition becomes the key provider for population on the ^5^I_7_ level; thus increasing pulse energy and shortening pulse duration of the ~3 μm pulse are helpful for decreasing the time delay. Alternatively, another pulsed pumping at 1.9 μm could be added which is located at the absorption band of ^5^I_8_ → ^5^I_7_ transition. This would result in faster population accumulation on the ^5^I_7_ level, thus providing the possibility of shortening the time delay. However, the time delay cannot be infinitely shortened due to the limitation of the ^5^I_6_ → ^5^I_7_ transition time.

Thirdly, we discuss the scheme in which the pulse durations and energies can be improved in this system. For the passively Q-switched ~3 μm components, the pulse duration was narrowed and the energy was improved with increasing pump power, and finally approached to constant values as when the SA became fully bleached. Like conventional Q-switched lasers, a higher modulation depth SA and shorter cavity length might be favored to obtain narrower and more energetic ~3 μm Q-switched pulses^55^. As the pump signal for ~2 μm gain switched components, higher energy of ~3 μm pulses is helpful for shortening the duration and improving the energy of ~2 μm pulses. In a similar way to the Q-switching, shorting the passive region i.e., free-space region also benefits to the narrowing of ~2 μm gain-switched pulses, which has been verified in previous experimental and theoretical demonstrations^24^,^56^. However, comprehensive optimization for all the cavity parameters including core size, fiber length, dopant concentration, etc., are required for further narrowing of pulse duration and improving of pulse energy.

## Conclusions

In this paper, an SESAM passively Q-switched induced gain-switched dual wavelength cascade pulsed fiber laser operating at wavelengths ~2 μm and ~3 μm has been demonstrated. As increasing pump power, the oscillator experienced different regimes: CW, stable ~3 μm Q-switching, stable ~3 μm Q-switching and stable ~2 μm gain switching with a half repetition rate as ~3 μm, stable ~3 μm Q-switching and unstable ~2 μm gain switching with jumping repetition rate, and finally stable ~3 μm Q-switching and stable ~2 μm gain switching with the same repetition rate as ~3 μm. A μs-level time delay between ~2 μm and its adjacent ~3 μm pulse was observed as a result of the time consumption for accumulating populations on the ^5^I_7_ level to arrive at the laser threshold level. Furthermore, some relevant variants, e.g., ~2 μm passively Q-switching induced ~3 μm gain switching based on a lower saturable influence SA at ~2 μm or passively Q-switching ~2 μm and ~3 μm pulses simultaneously based on a broadband SA, such as graphene, are expected to offer more flexibility for some practical applications. In addition, this result puts forward an universal approach for obtaining dual wavelength pulses in rare earth ions doped cascade transitions systems e.g., current well developed 976 nm pumped Er^3+^- and 1150 nm pumped Ho^3+^-doped ZBLAN fibers^11^,^14^, and future available 1710 nm pumped Dy^3+^-, 2040 nm pumped Pr^3+^-, 2950 nm pumped Tb^3+^-doped chacogenide fibers which correspond to far mid-IR emissions ranging from 3200 nm to 7500 nm^57^.

## Methods

### Experimental setup

**Figure 4:**
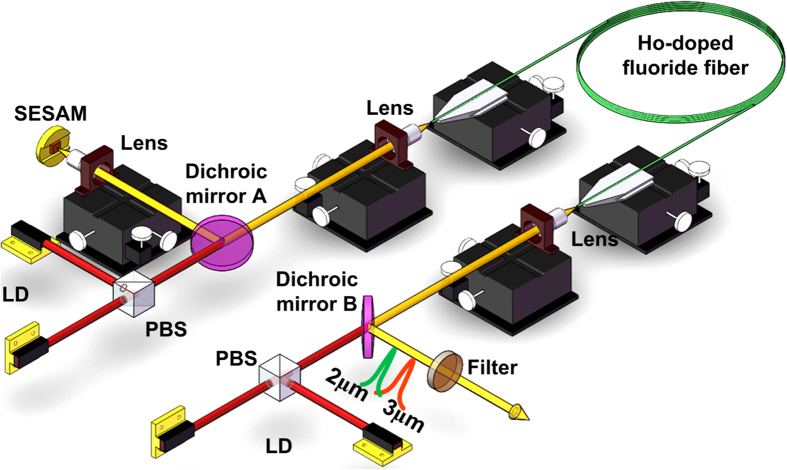
Schematic of the passively switched cascade dual wavelength Ho^3+^-doped fluoride fiber laser. The laser is pumped using four 1150 nm laser diodes (LD) coupled using two polarization beam splitters (PBS).

The schematic diagram of the passively switched dual wavelength cascade pulsed Ho^3+^-doped fluoride fiber laser is shown in Fig. 4. A two-end pumping method was employed to increase the pump power using two pairs of commercially available 1150 nm laser diodes (LDs) (Eagleyard Photonics, Berlin). Each pair of diode lasers was coupled into the gain fiber using polarization multiplexing via a polarized beam splitter (PBS) and focused using an anti-coated ZnSe objective lens (83%T@1150 nm, 70%T@~2 μm and ~3 μm)(Innovation Photonics, LFO-5-6-0.975/3 μm, 0.25 NA) with a 6.0 mm focal length. This lens also functions as the collimator for light coupled from the fiber core. Two identical dichroic mirrors (96%T@1150 nm, 95%R@ ~2 μm and ~3 μm) were respectively placed between the PBS and the ZnSe objective lens in each beam path at an angle of 45^0^ with respect to the pump beam to couple the generated ~2 μm and ~3 μm emissions. Specifically, the dichroic mirror A was used to direct the dual wavelength laser onto the SESAM InAs-based broadband SESAM (BATOP GmbH) via another ZnSe objective lens (<5%T@1150 nm, 80%T@~2 μm and ~3 μm). In this case, a reversed design is employed in the SESAM. The InAs saturable absorber layer is sandwiched between a bottom Au-coated mirror and an upper 625 μm GaAs wafer with an additional buffer layer to remove the negative influence of large lattice misfit. The Au-coated mirror has the advantage of broad operation band and excellent heat dissipation compared to conventional distributed Bragg reflector (DBR). At the upper surface of the GaAs wafer, an AR film for 3 μm was coated. In our previous work^23^, the nonlinear reflectivity of the SESAM was measured at 1 μm waveband due to lack of suitable mid-infrared pulsed source. Its modulation depth of 40.8%, non-saturable loss of 27.2% and saturation fluence of 1771 μJ/cm^-2^ were obtained by using the typical saturable absorption model to fit. In general, the ratio of modulation depth to non-saturable loss of the SESAM (i.e., 1.5 for our SESAM) mainly depends on the growth temperature and is independent of wavelength. Thus, this ratio can be used to estimate the modulation depths and non-saturable losses at other wavelengths. Specifically, the SESAM reflections were measured to be 47.3% and 52.2% using two lower power level CW ~2 μm and ~3 μm fiber lasers, respectively. According to the ratio of 1.5, the modulation depth and non-saturable loss were estimated to be 31.6% and 21.1% at ~2 μm and 23.3% and 15.5% at ~3 μm, respectively. Besides, some important parameters e.g., the relaxation time of ~10 ps, the damage threshold of 350 MW/cm^2^ and the saturable fluence of 70 μJ/cm^2^ at ~3 μm were also provided by the producer of the SESAM. The second dichroic mirror B was used to output the dual wavelength emissions. A ~3 μm bandpass filter (78%T@~3 μm) and a ~2 μm bandpass filter (81%T@~2 μm) were respectively placed along the output laser to remove the residual pump and separately extract the ~3 μm and ~2 μm emissions. The gain fiber in this case was a double clad fluoride fiber with a dopant concentration of 1.5 mol. % having a D-shaped pump core with a diameter of 125 μm across the circular cross section and a numerical aperture (NA) of 0.5, and a 10 μm core diameter with an NA of 0.2. The selected 7.0 m fiber length could provide ~95% pump absorption efficiency. In the experiment, the fiber end towards dichroic mirror B was perpendicularly cleaved and hence acted as both an output coupler and a cavity feedback with the aid of 4% Fresnel reflection. The other end of fiber was cleaved at an angle of 8^0^ to avoid parasitic lasing and constructing the single output scheme to ensure that most of laser radiation interacted with the SESAM. Thus, both the ~3 μm and ~2 μm emissions shared a common cavity can be terminated by the perpendicularly cleaved fiber on one end and the SESAM at the other.

### Measurement method

The output pulse trains were measured using an InAs photodetector with a response time of ~2 ns connected to a 500 MHz digital oscilloscope. A monochromator with a resolution of 0.1 nm (Princeton instrument Acton SP2300) was employed to measure the optical spectrum of the laser output radiation. An RF spectrum analyzer (Advantest R3267) with an adjustable resolution from 10 Hz to 100 MHz was connected to the same photodetector and used to measure the repetition rate and signal-to-noise ratio (SNR) of the output pulses.

## Additional Information

**How to cite this article**: Li, J. *et al.* Mid-infrared passively switched pulsed dual wavelength Ho^3+^-doped fluoride fiber laser at 3 µm and 2 µm. *Sci. Rep.* **5**, 10770; doi: 10.1038/srep10770 (2015).
